# Supplementary material for: WordCommentsAnalyzer: A windows software tool for qualitative research
Source: F1000Res. 2018 Sep 4;7:536. Originally published 2018 May 3. [Version 2] doi: 10.12688/f1000research.14819.2 (PMC6137411; doi:10.12688/f1000research.14819.2)
Supplement: Supplementary file 3 [file f1000research-7-17614-s0002.tgz › b2a79496-4ada-47c4-9682-a99ce867836d.zip!/Eight Iranian Health Journals/Journal of Knowledge and Health_41.docx]

© Journal of Knowledge and Health. This work is licensed under CC BY 4.0 (https://creativecommons.org/licenses/by/4.0/).

Volume & Issue: دوره 2، شماره 4: 1386

Title: محاسبه شاخص¬های حیاتی قلبی- عروقی افراد ایرانی با استفاده از معادلات مرجع

http://knh.shmu.ac.ir/index.php/site/article/view/252

مقدمه: افراد طبيعي اشخاصي هستند كه هيچ­گونه علایم بیماری قلبي- عروقي ندارند. اين افراد معمولاً داراي مقادير طبيعي هر كدام از شاخص­هاي حياتي قلبي، ريوي مي­باشند که تحت تأثير متغيرهايي مانند سن، جنس، قد و ژنتيك قرار مي­گيرند. هدف از اين تحقيق تعيين اندازه­هاي فيزيولوژيك شاخص­هاي قلبي- عروقي در جمعيت طبیعی ايران و به­دست آوردن معادلات مرجع براي اين گروه بود. مواد و روش­ها: افراد نمونه از شهرهاي مختلف انتخاب شده و پس از اطمينان از سلامت آن­ها در آزمايشگاه شاخص­هاي حياتي- قلبي شامل، ضربان قلب، فشار خون سيستولي، فشار خون دياستولي و فشار نبض با شيوه­هاي استاندارد و رعايت اخلاق پزشكي در جنس­هاي زن و مرد به­طور جداگانه اندازه­گیری شد. شرايط آزمايشگاه براي همه افراد يكسان و در حالت نشسته و پس از استراحت بوده است. نتايج: نتایج حاصل از اين اندازه­گيري­ها در گروه­هاي سني مختلف و به تفكيك زن و مرد تعيين شد. پس از تعيين ميانگين براي گروه­هاي سني و با استفاده از روش رگرسيون، معادلات مرجع براي هر كدام از شاخص­هاي حياتي مورد نظر به­شرح زير به­دست آمد: (قد) 132/0 - (سن) 086/0 - 791/101 = ضربان قلب (وزن) 15/0 + (جنس) 653/2 + (سن) 129/0 + 068/59 = فشار دیاستولی (وزن) 163/0 + (جنس) 293/4 + (قد) 64/0 + (سن) 454/0 + 078/82 = فشار سیستولی (جنس) 451/1 + (قد) 097/0 + (سن) 235/0 + 712/18 = فشار نبضنتیجه­گیری: با توجه به معادلات مرجع به­دست آمده براي جمعيت سالم ايران، مي­توان با در دست داشتن خصوصيات فرد مانند قد، وزن و سن، هر كدام از شاخص­هاي حياتي- قلبي را براي وي محاسبه و پيش­بيني نمود. به­عبارت ديگر ميزان متوسط استانداردي را كه براي فرد قابل تصور است به­دست مي­دهد، لذا اين مقادير مبناي مقايسه­اي براي طبيعي يا غيرطبيعي بودن شاخص­هاي اندازه­گيري شده در فرد است.

Title: بررسي وضعيت دريافت‌ ريزمغذي‌ها در بستگان درجه‌ يك‌ سالم، ديابتي و دچار اختلال تحمل گلوكز بيماران‌ ديابتي نوع II

http://knh.shmu.ac.ir/index.php/site/article/view/253

مقدمه: با توجه به نقش رژيم غذايي در افزايش خطر بروز ديابت نوع II، اين مطالعه با هدف تعيين وضعيت مصرف ريزمغذي‌ها در الگوي غذايي فاميل درجه يك بيماران ديابتي نوع II به انجام رسيده است. مواد و روش­ها: 210 نفر از افراد فاميل درجه يك بيماران ديابتي نوع II بر مبناي نتايج تست تحمل گلوکز به دو دسته مختل (بیماران ديابتي + بیماران دارای آزمون تحمل گلوکز مختل) و سالم تقسيم­بندي گرديدند. پس از تكميل پرسش­نامه ياد­داشت سه روزه خوراك در اين افراد، ميزان دريافت انرژي روزانه، منيزيم، روي، كلسيم، فسفر، كروميوم و ويتامين‌هاي B1 ,C ,E و B6 با استفاده از نرم­افزار N3 محاسبه و نتايج با آزمون t و سطح معني­داري 05/0 مورد مقايسه قرار گرفت. نتايج: میانگين سن در دو گروه مختل و سـالم به­ترتيب 2/5 ± 9/48 و 7/6 ± 6/43 سال (001/0= P) و میانگین نمایه توده بدنی در دو گروه نیز  7/0 ± 3/29 و 8/0 ± 7/28 كيلوگرم بر مترمربع بود. ميانگين دريافت انرژي، منيزيم، روي، كلسيم، فسفر، كروميوم و ويتامين‌هاي E، C، B1 و B6 در دو گروه تفاوت معني‌داري نداشت (05/0<P).نتيجه­گيري: هرچند تفاوتي بين دريافت ريزمغذي‌ها در دو گروه مشاهده نشده است ولي مقادير دريافت بعضي از ريزمغذي‌ها مانند كروميوم، منيزيم، روي و ويتامين‌ E كم­تر از مقادير توصيه شده مي­باشد. اهميت اين مطلب در

Title: بررسي عوارض ديابت در بيماران مراجعه¬كننده به كلينيك ديابت شهر شاهرود

http://knh.shmu.ac.ir/index.php/site/article/view/254

مقدمه: امروزه ديابت يكي از مهم­ترين مشكلات بهداشتي- درماني و اقتصادي- اجتماعي جهان محسوب مي­شود به­گونه­اي كه بيش از صد و هفتاد ميليون نفر در دنيا مبتلا به اين بيماري هستند. در كشور ما بالغ بر چهار ميليون نفر به این بيماري مبتلا هستند. افراد ديابتي بيش­تر از افراد عادي در معرض عوارضی چون نابينايي، حملات قلبي، نوروپاتي، نفروپاتي، بيماري­هاي اندام تحتاني و قطع عضو هستند. لذا با توجه به اهميت شيوع ديابت و عوارض آن تصمیم گرفته­شد كه فراوانی انواع ديابت و عوارض آن را در بيماران ديابتي مراجعه­كننده به كلينيك ديابت شهر شاهرود بررسي گردد تا با استفاده از نتايج اين تحقيقات بتوان با پيش­گيري مناسب از عوارض ديابت تا حد امكان جلوگيري كرد. مواد و روش­ها: پژوهش حاضر يك مطالعه توصيفي مي­باشد كه بر روي 340 بيمار ديابتي مراجعه­كننده به كلينيك ديابت انجام شد. اطلاعات مورد نياز از طريق پرسش­نامه و با توجه به موارد ثبت شده جمع­آوري و داده­های مورد نظر پس از ورود به نرم افزار  SPSSبا استفاده از آزمون­هاي c2 و t مستقل مورد تجزيه و تحليل قرار گرفت. داده­های کیفی با فراوانی و درصد فراوانی و داده­های کمی با میانگین و انحراف معیار بیان گردید. نتایج: از مجموع 340 بيمار ديابتي، 240 نفر زن و 100 نفر مرد بودند. ميانگين سني بيماران 2/15 ± 2/50 سال بود. 9/87%‌ بيماران ديابت نوعII و 8/11% ديابت نوع I و 3/0% ديابت دوران بارداري داشتند. میانگین مدت ابتلاء به ديابت در بیماران 5/5 سال بود. هم­چنين مدت ابتلا به ديابت در 2/51% بيماران کم­تر از 5 سال بود.‌ 4/77% از مبتلايان به ديابت تحصيلات كم­تر از ديپلم داشتند. بين ديابت نوعII  و شاخص نمایه توده بدن و تحصيلات نيز ارتباط معني­داري مشاهده گرديد. نتايج بيانگر آن است كه سابقه فاميلي مثبت در 1/44 % بیماران مبتلا به ديابت نوع دو گزارش شده است. در بررسي عوارض ديابت مشاهده شد كه 3/77% از بيماران مبتلا به نوروپاتي، 9/13% نفروپاتي، 5/33%‌ رتينوپاتي، 8/21% ‌اختلالات گوارشي و 7/12%‌ مشكلات در اندام­ها (زخم پا، آمپوتاسیون اندام تحتانی و ...)، 6/18% بيماري قلبي– عروقی و 9/5 % گواتر داشتند. نتيجه­گيري: نتايج حاكي از شيوع بالاي عوارض و عوامل خطر در بیماران دیابتی مي­باشد. لذا براي كاهش عوارض، نياز به آموزش همگاني از طريق رسانه­ها و آموزش چهره به چهره در افراد مراجعه­كننده به كلينيك ديابت ضروري به­نظر مي­رسد تا از مشكلات بيماران، خانواده­ها و جامعه كاسته شود.

Title: بررسی میزان به¬کارگیری داروهای گیاهی در زنان مراجعه¬کننده به مراکز بهداشتی- درمانی شهرکرد

http://knh.shmu.ac.ir/index.php/site/article/view/256

مقدمه: با توجه به تأثیر جدی درمان­های دارویی بر سلامت مردم، استفاده معقول و منطقی از داروها اعم از گیاهی و شیمیایی بخش مهمی از سیاست­های بهداشتی و درمانی جامعه را تشکیل می­دهد. نظر به استقبال جامعه زنان در به­کارگیری توأم گیاه درمانی با درمان­های دارویی و یا جایگزین نمودن مصرف داروهای گیاهی جهت بسیاری از بیماری­ها حتی در دوران بارداری، در این مطالعه میزان به­کارگیری داروهاي گياهي در زنان شهرکرد صورت گرفت. مواد و روش­ها: در اين بررسي توصيفي مقطعي، 1449 زن مراجعه­کننده به مراکز بهداشتی و درمانی مورد پرسش قرار­گرفتند. پرسش­نامه مبتني بر هدف تهيه و از طريق مصاحبه حضوري تکميل گرديد. روش نمونه­گيري آسان و آزمون­هاي آماري مورد استفاده شامل آزمون c2 و t  می­باشند. نتایج: ميزان استفاده از داروي گياهي در اين مطالعه 3/68 درصد بوده و 3/85 درصد واحدهاي پژوهش نگرش مثبتي نسبت به داروهاي گياهي داشتند. 6/62 درصد افراد معتقد بودند داروی گیاهی هیچ­گونه عارضه­ای در بر ندارد. در این مطالعه، زنان از 126 گونه گیاهی برای درمان 101 مشکل استفاده نمی­کردند. شايع­ترين علت مصرف داروي گياهي سرماخوردگي (47/29 درصد) و شايع­ترين گياه مورد استفاده بومادران بود. 60 درصد افراد گزارش نمودند با مصرف داروی گیاهی بیماری آنان کاملاً درمان شده است. 4/43 درصد افراد به­طور همزمان از داروی گیاهی و سنتتیک و 62 درصد آنان ترکیبی از گیاهان را در هر بار مصرف استفاده کرده­اند. 4/62 درصد زنان مورد مطالعه معتقد بودند مصرف هم­زمان گیاه با دارو ضرر ندارد یا در این زمینه اظهار بی­اطلاعی کردند. 8/7 درصد افراد گزارش کردند با مصرف داروهای گیاهی دچار عوارض شدند. 9/55 درصد افراد مصرف داروي گياهي را به پزشک معالج خود اطلاع نداده بودند که شايع­ترين علت عدم اطلاع به پزشک را طبيعي و بي­ضرر بودن گياه (9/39 درصد) گزارش کردند. در اين بررسي افرادي که درآمد و تحصیلات کم­تر و سن بين 20 تا 29 سال داشتند بيش­تر از داروي گياهي استفاده می­کردند اما اين ارتباط از نظر آماري معني­دار نبود. بين ابتلا به بیماری مزمن و مصرف گياه ارتباط آماري معني­دار وجود داشت) 0001/0(P<. نتيجه­گيري: استفاده گسترده از داروهای گیاهی توسط زنان و عدم آگاهی آنان از عوارض جانبی، دوز داوها، دوز سمی، تداخلات داروهای گیاهی، تداخلات داروهای گیاهی با داروهای شیمیایی و... نیاز همگانی جامعه به آموزش خاص به پزشکان به­عنوان اصلی­ترین مرجع بیماران و نیز سایر کارکنان مراکز بهداشتی، درمانی درخصوص گیاهان دارویی به­ویژه پر مصرف را به­وضوح آشکار می­سازد.

Title: تعيين ميزان تكرارپذيري تشخيصي انواع هيپرپلازي‌هاي آندومتر و كارسينوم تمايز يافته اندومتريوئيد بر اساس يافته‌هاي مورفولوژيك و مقايسه آن با نتايج آناليز مورفومتريك كامپيوتري

http://knh.shmu.ac.ir/index.php/site/article/view/257

مقدمه: ارزيابي، تشخيص و درمان مناسب ضايعات پیش­سرطانی آندومتر در طي ساليان دراز هم­چنان در بين آسیب­شناسان و متخصصین زنان به­صورت يك مشكل باقي مانده است. اين موضوع كه بعضي از هيپرپلازي‌ها، هم­چون انواع سرطان­ها از نظر فنوتیپ منوكلونال مي‌باشند، به­صورت وسيله‌اي مفيد در تشخيص آن­ها شناخته شده است. جهت جدا نمودن ضايعات منوكلونال از پلي­كلونال، آناليز مورفومتريك كامپيوتري در طي مطالعات متعدد روش مناسبي معرفي گرديده است. مواد و روش­ها: در این مطالعه 100 نمونه انواع هيپرپلازي‌هاي آندومتر با مراجعه به بخش پاتولوژی بیمارستان میرزا کوچک­خان تهران انتخاب و ابتدا از نظر تكرارپذيري تشخيصی بين فردی و درون فردي و سپس 55 مورد آن­ها از نظر آناليز مورفومتريك بررسي شدند. نمونه­ها در فرمالدئید 10% فیکس شده و در پارافین قالب­گیری و برش­های H & E استاندارد از آن­ها تهیه شده بود. سپس با استفاده از کامپیوتر آنالیز مورفومتریک انجام گردید و سه شاخص درصد حجمی استروما (VPS)، چگالی سطح خارجی (Out SD) و انحراف معیار کوچک­ترین محور هسته (SDSNA) محاسبه و بر مبنای آن­ها D- score تعیین گردید. نتایج: تشخيص هایپرپلازی ساده (SH) و کارسینوم تمایز­یافته آندومتریوئید (WDA) در بين آسیب­شناسان تكرارپذيري خوبي داشت، ولي هيپرپلازي­های کمپلکس  CHو آتیپیکال (AH) از تكرارپذيري كمي برخوردار بود. تكرارپذيري درون فردي در حد عالي بود. با آناليز مورفومتريك ضايعات به گروه­هاي 55% < VPS ، 1D-score < و 55% VPS ≥ ، 1D-score ≥ و يا به­عنوان ديگر منوكلونال و پلي­كلونال تقسيم شدند. با مقايسه نتايج آسیب­شناسان مختلف با نتايج VPS و D-score مشاهده شد كه آسیب­شناس با سابقه كاري بيش­تر در ژنيكوپاتولوژي به­خوبي توانسته بود ضايعات گروه­هاي مختلف را مطابق با نتايج VPS و D-score از یکدیگر جدا نماید، ولي آسیب­شناسان با تجربه­ی كم­تر در ژنيكوپاتولوژي نتوانسته بودند به­خوبي بر اساس ديد مورفولوژي آن­ها را از هم جدا كنند.نتیجه­گیری: بر اساس نتايج آناليز مورفومتريك ضايعات هيپرپلازي راحت­تر به دو گروه هیپرپلازی آندومتر (EH) و نئوپلاسم داخل اپی­تلیالی آندومتر (EIN) تقسيم مي‌شوند و تكرارپذيري و صحت تشخيصي نسبت به ديد ذهنی آسیب­شناسان در حد بالاتري قرار مي‌گيرد و میزان درمان بیش­تر و کم­تر از حد مورد نیاز کاهش می­یابد.

Title: تأثیر روان درمانی راه حل محور بر رضایت زناشویی در زوج¬های هر دو شاغل

http://knh.shmu.ac.ir/index.php/site/article/view/279

مقدمه: درمان راه حل محور یکی از انواع درمان­های کوتاه مدت است که بر یافتن راه حل­ها توسط مراجع با کمک درمان­گر تأکید دارد. پژوهش حاضر به­منظور بررسی اثر روان درمانی راه حل محور بر رضایت زناشویی زوج­های هردو شاغل صورت گرفته است. مواد و روش­ها: در این پژوهش از بین زوجین فرهنگی هر دو شاغل که حاضر به همکاری بودند پس از شرکت در پیش آزمون، 24 زوج انتخاب و به­صورت تصادفی در دو گروه آزمایش و کنترل قرار گرفتند که گروه آزمایش 7 جلسه تحت درمان قرار گرفت. ابزار پژوهش عبارت است از پرسش­نامه­ی رضایت زناشویی فرم تجدید نظر شده MSI-R که توسط اشنایدر ساخته شده است. برای تجزیه و تحلیل داده­ها علاوه بر استفاده از روش­های آمار توصیفی از آزمون تحلیل واریانس کرت­های خرد شده استفاده شده است. نتایج: متغیر­های مورد نظر (پرخاشگری- زمان با هم بودن- توافق درباره مسایل مالی– رضایت جنسی) با توجه به آزمون تحلیل واریانس کرت­های تقسیم شده و 01/0>P مورد تجزیه و تحلیل قرار گرفتند که در هر چهار متغیر تفاوت معنی­داری در سطوح مختلف متغیر­های آزمون (پیش آزمون– پس آزمون) مشاهده شد . نتیجه­گیری: از نتایج چنین به­نظر می­رسد که روان درمانی راه حل محور می­تواند پرخاشگری و مشکلات جنسی زوج­ها را کاهش داده و باعث بهبود زمان با هم بودن و توافق درباره مسایل مالی آن­ها شود.

Title: بررسی تأثیر آموزش بر آگاهی، نگرش و عملکرد دانش¬آموزان دختر مقطع راهنمایی در مورد مصرف شیر و لبنیات

http://knh.shmu.ac.ir/index.php/site/article/view/280

مقدمه: تغذیه متعادل و استفاده از گروه شیر و لبنیات از مبانی مهم سلامتی کودکان و رشد جسمی و افزایش بازدهی آموزشی می­باشد و آموزش یکی از روش­های افزایش آگاهی و ایجاد نگرش و عملکرد صحیح در دانش­آموزان است. این مطالعه با هدف بررسی میزان تأثیر آموزش بر آگاهی، نگرش و عملکرد دانش­آموزان دختر مقطع راهنمایی شهرستان یزد در مورد مصرف شیر و لبنیات انجام پذیرفته است. مواد و روش­ها: در این مطالعه نیمه تجربی، چهار کلاس دوم راهنمایی (106 دانش آموز) که از لحاظ تحصیلات و شغل والدین و بعد خانوار هم­گون بودند انتخاب و به­طور تصادفی به دو گروه مورد (دو کلاس با 52 دانش آموز) و شاهد (دو کلاس با 54 دانش آموز) تقسیم شدند. ابزار گردآوری اطلاعات پرسش­نامه­ای شامل چهار قسمت بود: سؤالات زمینه­ای و سؤالاتی در مورد آگاهی، نگرش و عملکرد دانش­آموزان در مورد مصرف شیر و لبنیات که با توجه به مطالعات قبلی طراحی شد. این پرسش­نامه به­صورت پیش آزمون (Pre-test) در هر دو گروه تکمیل گردید. سپس بر اساس اطلاعات به­دست آمده، برنامه آموزشی برای گروه مورد طراحی و اجرا شد و پس از دو ماه آزمون ثانویه (Post-test) از هر دو گروه به­عمل آمد. داده­ها بعد از کدگذاری با استفاده از نرم افزار SPSS و آزمون آماری t زوجی تجزیه و تحلیل شد. نتایج: بر اساس نتایج آزمون t زوجی، امتیاز آگاهی و نگرش دانش­آموزان گروه مورد بعد از آموزش به­طور معنی­داری افزایش یافته بود (001/0=P) ولی میزان افزایش امتیاز عملکرد آن­ها معنی­دار نبود (06/0=P). در گروه شاهد هیچ تغییر معنی­داری در نگرش (11/0=P) و عملکرد (68/0=P) آن­ها مشاهده نشد. ولی نمره آگاهی گروه شاهد به­طور معنی­داری افزایش یافته بود (001/0=P). با این وجود میزان افزایش آگاهی گروه مورد به­طور معنی­داری بیش­تر از گروه شاهد بود (001/0=P). نتیجه گیری: برنامه آموزش تغذیه در مورد مصرف شیر و لبنیات در ارتقاء دانش، نگرش و رفتار تغذیه ای دانش­آموزان تحت مطالعه تأثیر مثبت داشته است. با توجه به پایین بودن سطح آگاهی، نگرش و عملکرد دختران و برای بهبود و اصلاح رفتار تغذیه­ای آنان در مورد مصرف شیر و لبنیات استفاده از برنامه آموزشی ضروری به­نظر می­رسد.

Title: بررسی وضعیت ایمنی و ارگونومیک منازل سالمندان بر اساس نظریه هم¬سازی انسان- محیط در کشور مالزی

http://knh.shmu.ac.ir/index.php/site/article/view/281

مقدمه: سالمندی پدیده­ای جهانی در دنیای امروز به­حساب می­آید. در کشورهای آسیایی من­جمله مالزی جمعیت سالمندان رو به فزونی است. به موازات افزایش سن متوسط جامعه و گسترش سالمندی و مشکلات متعدد سالمندان، لزوم اصلاح و به­سازی محیط زندگی آنان مطرح می­گردد. بر اساس نظریه هم­سازی انسان– محیط، سالمندان باید در محیطی زندگی نمایند که بیش­ترین هم­سازی را با محیط خود داشته باشند. چنین محیطی دارای کم­ترین عوامل خطر محیطی بوده و موجب ارتقاء کیفیت زندگی ایشان می­گردد. مواد و روش­ها: در این پژوهش 386 نفر از سالمندان در مناطق شهری کشور مالزی به صورت تصادفی انتخاب شدند. با استفاده از مصاحبه رو در رو، برگ مشاهده بازدید منازل و پرسش­نامه رضایت سالمندان از ایمنی منزل، اطلاعات جامعی از وضعیت ایمنی و ارگونومی محیط زندگی سالمندان کسب شد. نتایج: بر اساس نتایج به­دست آمده از 6 موقعیت اتاق خواب، اتاق نشیمن، پله­ها، آشپزخانه، توالت و حمام، علل متعدد سوانح در هر یک از این محل­ها تعیین گردیدند. مهم­ترین و شایع­ترین این علل در بازدید از منازل شامل کوچک بودن فضا، ناهموار بودن کف، لغزندگی کف، کمبود نور و ... بودند. نتیجه گیری: ارتقاء کیفیت و رضایت سالمندان از زندگی و محیط منزل با تأمین هم­سازی انسان– محیط در سالمندان میسر است. استفاده از دستگیره­های مناسب در، کف­پوش­های غیر لغزنده، تعبیه میله کنار در حمام، توالت، راه­روها، پله­ها و اتاق­ها، رفع هرگونه ناهمواری در کف و ارتقاء استقلال در زندگی فردی سالمند موجب تأمین ایمنی و رضایت بیش­تر برای سالمندان می­گردد.
